# Catheter-based Therapy for Massive Pulmonary Embolism in an Elderly Woman with Chest Pain and Dyspnea: Case Report

**DOI:** 10.7759/cureus.5771

**Published:** 2019-09-26

**Authors:** Hugo R Ramos, Maria Soledad Ceballos, Hernan Alvarenga, Eduardo C Conci, Carlos S Balestrini

**Affiliations:** 1 Division of Cardiology, Instituto Modelo De Cardiologia, Cordoba, ARG; 2 Division of Cardiology, Instituto Modelo De Cardiología, Cordoba, ARG

**Keywords:** acute, pulmonary embolism, treatment, catheter-based, elderly

## Abstract

An 80-year-old woman presented with chest pain and dyspnea. The electrocardiogram (ECG) showed a known chronic complete left bundle branch block and elevated levels of high-sensitivity cardiac Troponin T. The first diagnosis was acute coronary syndrome, but a few hours later she developed shock and syncope; after resuscitation a coronary angiography was performed but it did not show any acute coronary obstruction. The echocardiogram showed McConnell’s sign suggesting a massive pulmonary embolism; the pulmonary angiography showed large thrombi in both branches of pulmonary artery, so a catheter-based treatment was performed with thromboaspiration and rt-PA administration, and a significant improvement of blood pressure, clinical condition and right ventricle function was observed. In spite of bleeding at the puncture sites (femoral artery and vein), controlled by local compression, catheter-based therapy in massive pulmonary embolism was associated with survival and satisfactory outcome. A combined fragmentation/thromboaspiration and catheter-directed fibrinolysis strategy may be useful to reduce the embolic load, improve RV function, and reduce mortality.

## Introduction

Chest pain and dyspnea are frequent symptoms in elderly people who attend to the emergency department (ED); but the mortality rate is high for patients with acute coronary syndrome (ACS) and acute pulmonary embolism (PE) if not properly diagnosed and treated [1]. Frequently, acute chest pain is assumed as coronary in origin, especially if it is associated with high levels of cardiac troponin, but there are a lot of causes of its elevation, one of them being PE [2]. PE is recurrent and its symptoms and signs are non-specific at the onset, but rapidly the clinical course may run from no-high risk to high-risk with acute *cor pulmonale*, shock, and death [3]. The treatment of severe PE consists in systemic fibrinolysis, catheter-based therapy, or surgical embolectomy to reduce clot burden, reducing the pulmonary artery pressure and the dysfunction of the right ventricle [3]. A variety of devices are being used for catheter-based therapy, but their efficacy and security are not completely proven, and different centers are using different devices and techniques. In this case we report an old woman with an initial diagnosis of ACS but the right one was severe PE, which was treated successfully with catheter-based thromboaspiration and fibrinolysis with 8F catheter in both branches of the pulmonary artery.

## Case presentation

**Figure 1:**
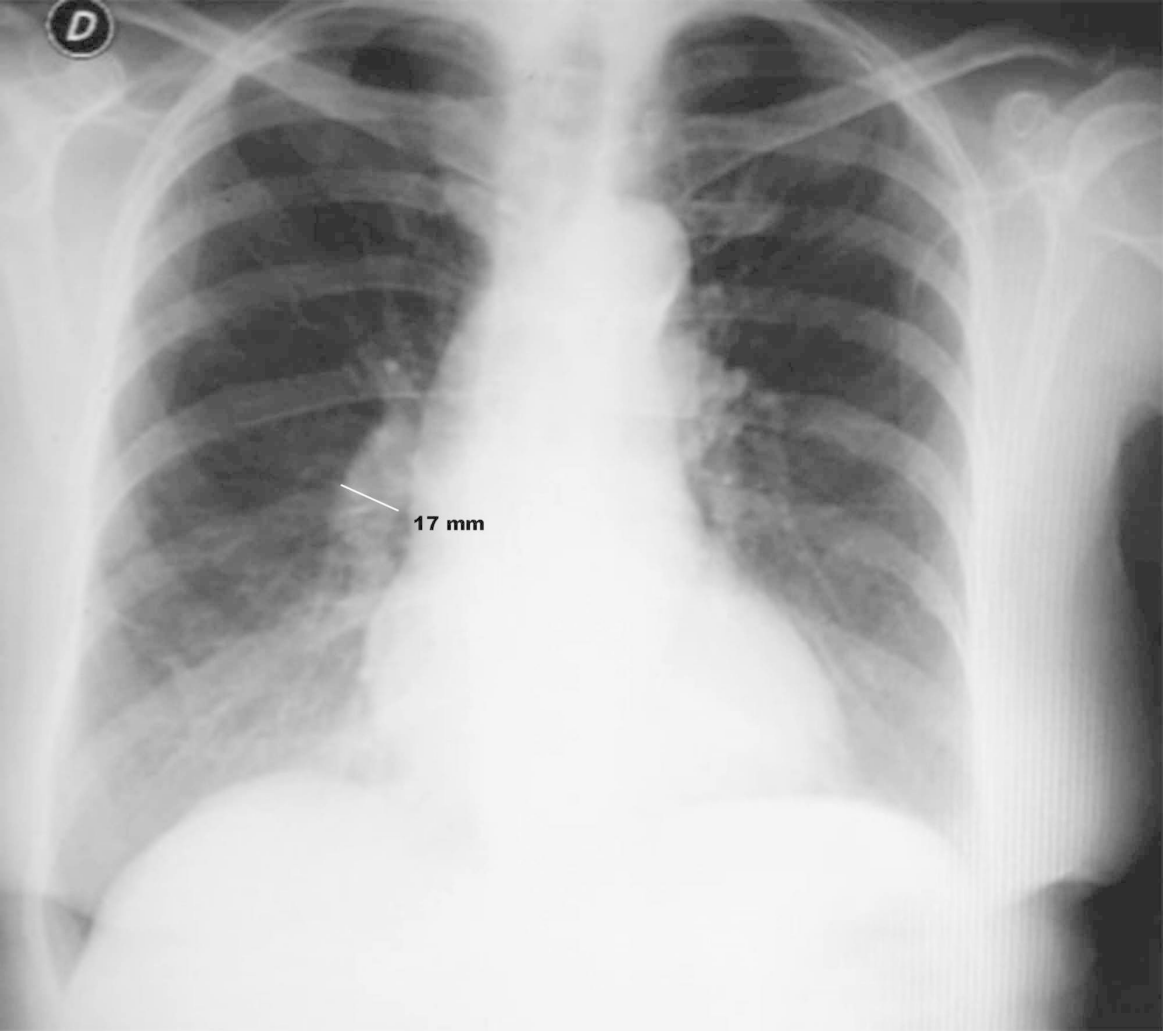
Chest X-ray on admission. The first interpretation was a near-normal radiograph with chronic findings (see text), but there is a subtle enlargement of the descending right pulmonary artery (diameter 17 mm).

An 80-year-old woman presented with dyspnea and atypical chest pain of recent onset. Physical examination in the ED revealed blood pressure (BP) 136/90 mmHg, heart rate 100 bpm, respiratory rate 28 per min, Tº 36, 1º C, Sat O_2_ 95% with room air, a 2/6 ejection systolic murmur in the aortic area, with no other positive findings (no edema, jugular distension, or pulmonary rales were found). She had a history of smoking, hypertension, and hypercholesterolemia. The electrocardiogram (ECG) showed a known chronic complete left bundle branch block (LBBB), and the measured hs-cTnT was 204 ng/L (URL percentile 99th: 14 ng/L). The chest X-ray showed aortic sclerosis, slight fibrotic changes at the left lung base with no other significant abnormalities apparently, but subtle enlargement of the descending right pulmonary artery was not seen (Figure 1).

**Figure 2:**
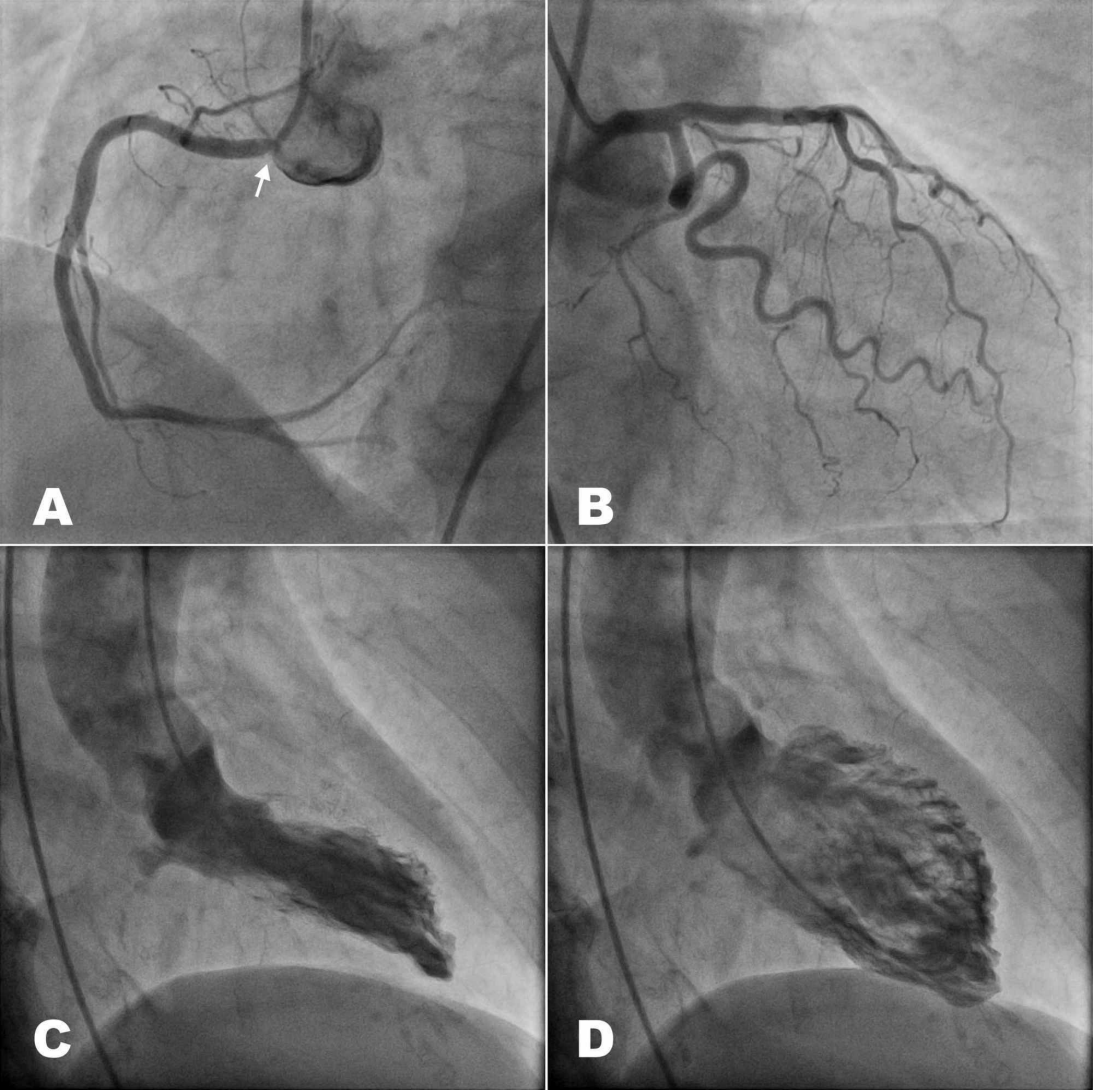
Coronary angiography. (A) Left anterior oblique view of right coronary artery with calcified obstruction of 80% in the ostium (arrow). (B) Right anterior oblique view with non-significant lesions in left main, left anterior descending artery (30% in middle third) and left circumflex artery. (C) Left ventricle in systole (right oblique view). (D) Left ventricle in diastole (right oblique view).

The diagnosis was ACS without ST-segment elevation with LBBB and, although heart failure and PE were considered, the decision was to start treatment with aspirin, clopidogrel, bisoprolol, nitroglycerin, and enoxaparin. However, three hours later, she suddenly experienced syncope with systolic BP 70 mmHg, and bradycardia 30 bpm, for which she needed atropine and inotropes to recover. A coronary angiography was performed via right femoral artery access, showing a calcified obstructive lesion of 80% in the ostium of the right coronary artery, and an obstruction of 30% in the middle third of the anterior descending artery. The left main and the circumflex artery did not present any obstructions. The left ventriculogram showed apical hypokinesia with slight systolic dysfunction, cardiac output 2.85 L/m^2^, cardiac index 1.54 L/min/m^2^, wedge pressure 12 mmHg, and pulmonary artery pressures 64/10/35 mmHg (Figure 2A-2D).

Coronary lesions were not considered acute and an echocardiogram was performed which showed the following: right atrial dilation and right ventricle (RV) dilation with septal flattening and marked hypokinesia at the basal and mid segments of the RV, with normal apical motility (McConnell's sign), severe tricuspid regurgitation, and a systolic pulmonary artery pressure of 72 mmHg (Video 1).

**Video 1:** Echocardiogram suggestive of severe pulmonary embolism. Four-chamber view: right ventricle enlargement, free wall hypokinesis, with sparing of the apex (McConnell's sign); interventricular septal flattening and mild pericardial effusion.

**Figure 3:**
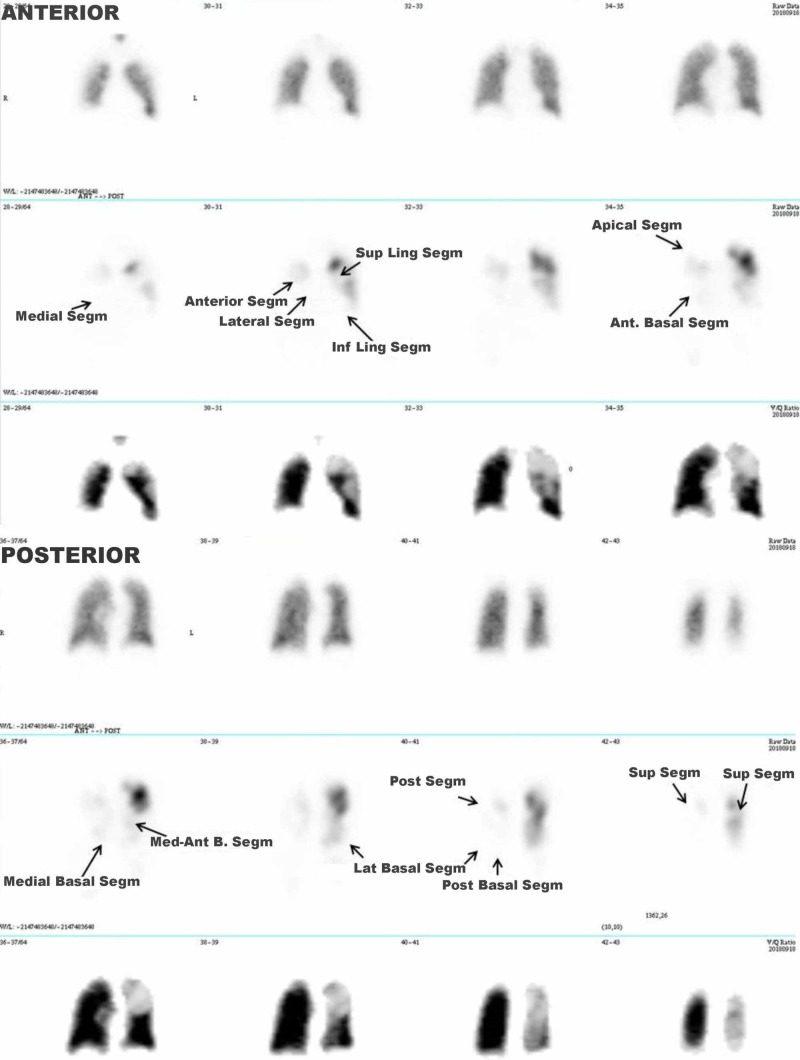
Pulmonary radionuclide V/Q scintigraphy. Normal ventilation with multiple perfusion defects: severe defect of perfusion of right lung, and segmental and subsegmental defects of perfusion in the left lung (arrows). Ant.: anterior; B.: basal; Inf: inferior; Lat.: lateral; Ling.: lingula; Med-Ant.: medial-anterior; Post.: posterior; Segm.: segment; Sup.: superior.

The left ventricle (LV) had mild abnormal motility similar to those found in the angiography and the LV ejection fraction (LVEF) was 51%. McConnell’s sign is independent of left ventricle, just from right ventricle related to pulmonary embolism, and mild hypokinesia of left ventricle possibly related to a previous unknown ischemia. The D-Dimer test was 4,149 μg/L (age-adjusted cut-off: 800 μg/L), and an NT-proBNP concentration of 1,106 ng/L. A pulmonary radionuclide V/Q scintigraphy was performed, which showed exclusion of the right lung, and segmental and subsegmental perfusion defects in the left lung affecting 81% of the pulmonary vascular bed (Figure 3).

**Figure 4:**
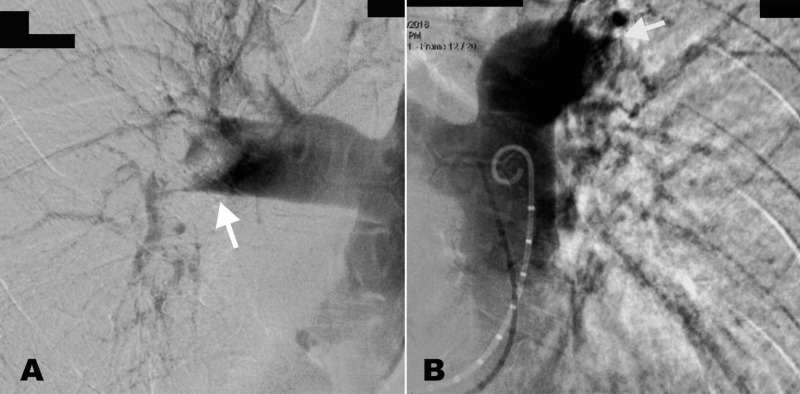
Diagnostic pulmonary angiography. (A) Right pulmonary artery with a large thrombus with a concave edge (arrow). (B) Left pulmonary artery with a thrombus in the lower left branch (arrow).

The pulmonary angiography showed a large distal occlusive thrombus in the main right pulmonary artery and a large mobile thrombus in the lower lobe branch of the left pulmonary artery (Figure 4A-4B). Creatinine before the angiogram was 0.97 mg/dL, and three days after was 0.79 mg/dL. It remained stable after seven days and 30 days.

**Figure 5:**
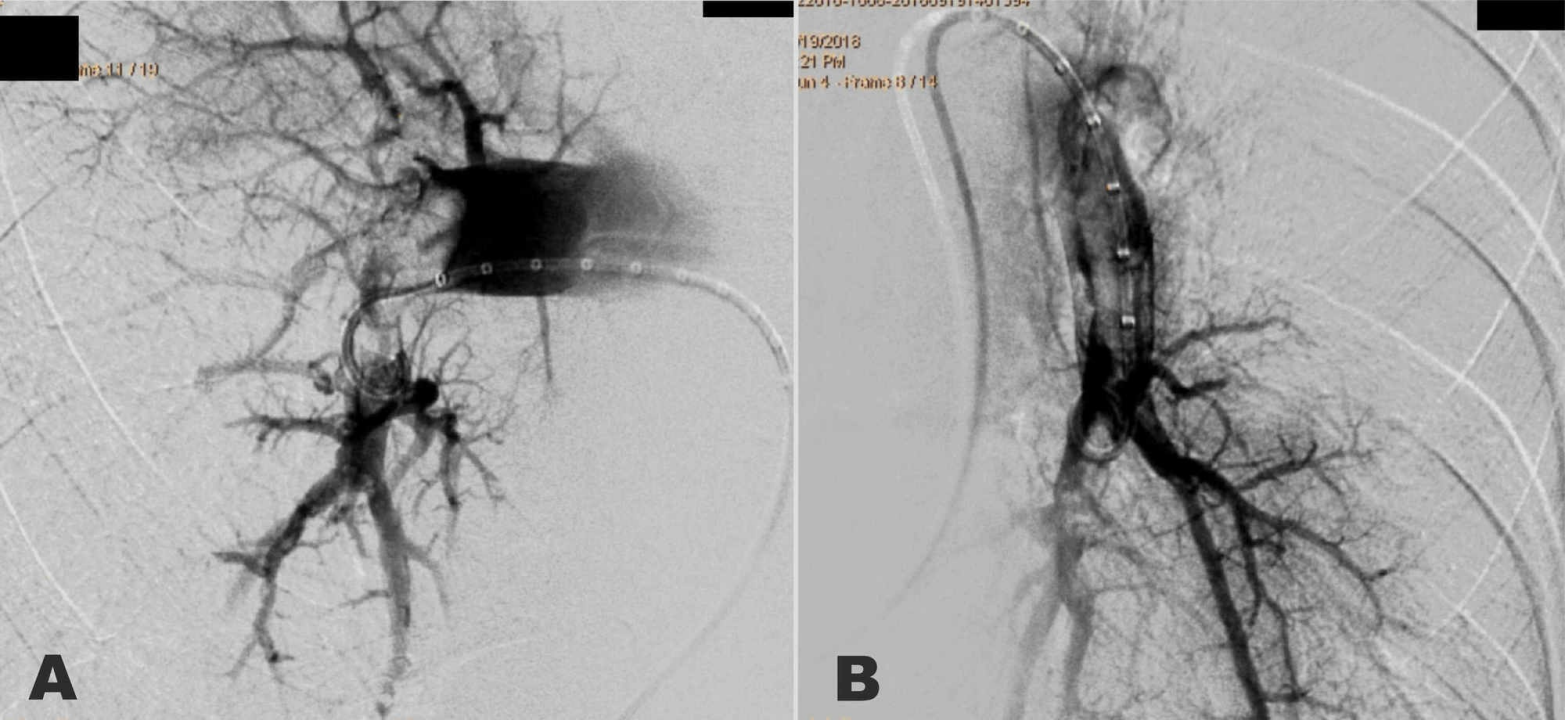
Catheter-based treatment. (A) Right pulmonary artery after initial thromboaspiration. (B) Lower left branch of pulmonary artery after thromboaspiration. Both of them with significant reperfusion.

**Figure 6:**
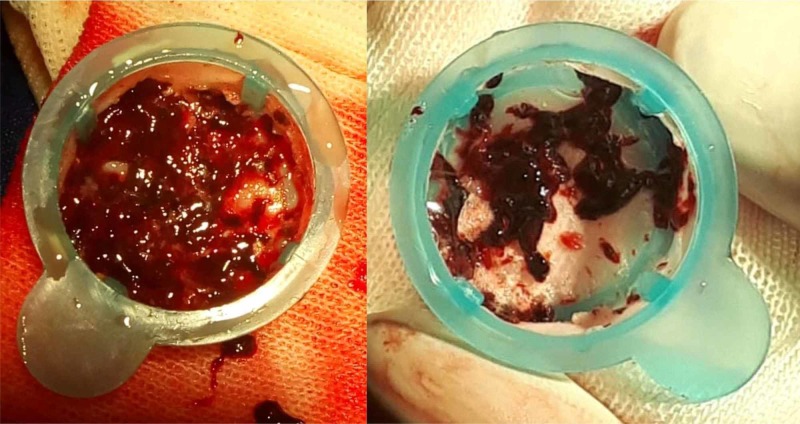
Pulmonary catheter-based embolectomy. Multiple emboli recovered from right and left pulmonary arteries after mechanical clot fragmentation and aspiration.

An immediate fragmentation of the emboli was performed together with catheter aspiration using a diagnostic 8F FR3 catheter of right coronary artery (Cordis, USA) (Figure 5A-5B), extracting multiple clots with immediate reperfusion (Figure 6).

**Figure 7:**
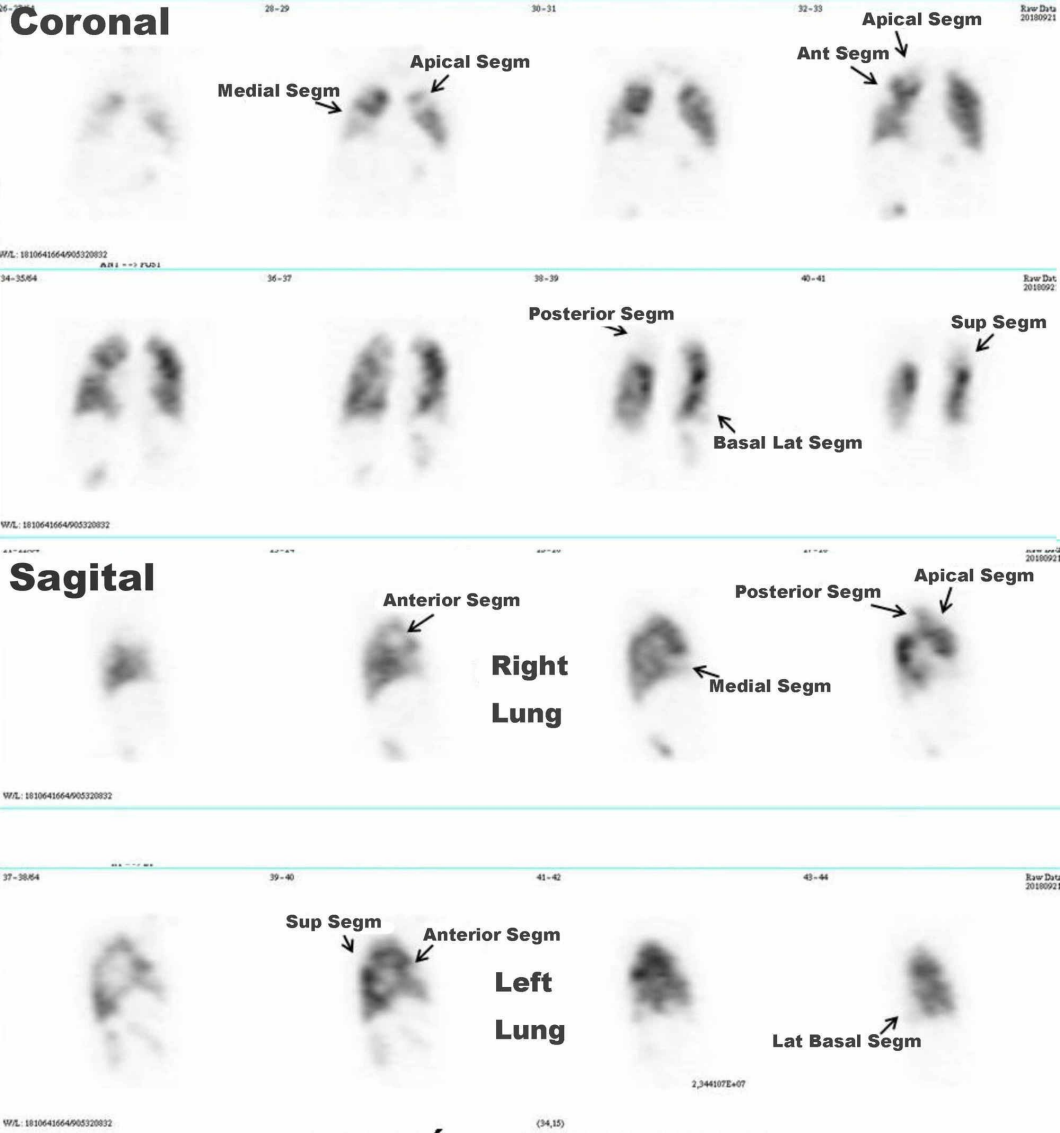
Pulmonary radionuclide perfusion scintigraphy. Significant improvement of perfusion defects 48 hours after catheter-based thromboaspiration and fibrinolysis treatment, especially in the right lung. Abbreviations as in Figure 3.

This was followed by rt-PA infusion into the right pulmonary branch (15 mg) and into the left pulmonary branch (10 mg) guided by 8F catheters; in addition, 30 mg of rt-PA were intravenously administered within three hours. The extra dose of 5 mg r-tPA to right pulmonary branch was delivered due to the embolic burden remained after the infusion of 10 mg. The patient experienced a clinical improvement with BP 100/60 mmHg, HR 78 bpm, Sat O_2_ 88% with oxygen mask and 92% with room air 24 hours later, but there was bleeding from the femoral vein and arterial puncture sites which was stopped with local compression and discontinuation of the rt-PA infusion treatment. After 48 hours, a new pulmonary perfusion radionuclide scintigraphy was performed, which showed significant amelioration, with perfusion defects in only 28% of the pulmonary vascular bed (Figure 7).

A Doppler ultrasound of the lower limbs was performed, which did not show deep venous thrombosis, and after five days the patient was discharged with apixaban. She evolved with mild anemia, and was clinically stable. Thirty days later, she was on class I, and a new echocardiogram showed normal RV size and function, with a systolic pressure of pulmonary artery of 26 mmHg.

## Discussion

At the beginning, the PE may go unnoticed since some of its manifestations may be confused with an ACS, especially in elderly patients [1]. The clinical severity may be assessed as massive, submassive or low-risk PE [3], or validated scores can be used to predict death at 30 days, such as the Pulmonary Embolism Severity Index (PESI) [4] or the simplified PESI (sPESI) [5]. In this patient, the initial evolution indicated an intermediate-to-low-risk PE but the embolic recurrence recategorized it as a high-risk PE due to the presence of shock, RV dysfunction, and biomarkers elevation (hs-cTnT and NT-proBNP) [3,6]. Treatment for severe PE requires systemic fibrinolysis or catheter-directed fibrinolysis using less dose, mechanical thrombectomy/thromboaspiration, or both [3,6]. In this case, the recent femoral artery access for the coronary angiography and the age (over 75) were discussed at the moment of making a decision, but finally, a thrombi fragmentation and catheter aspiration strategy were chosen together with a catheter-directed rt-PA administration into both branches of the pulmonary artery [3,7]. The procedure brought about a speedy and significant amelioration of the clinical condition, despite controllable bleeding from the venous and arterial puncture sites; this clinical amelioration correlated objectively with the perfusion improvement observed immediately after in the pulmonary angiography and after 48 hours in the pulmonary radionuclide perfusion scintigraphy. The major risk of systemic fibrinolysis is hemorrhage, which increases four times for people over 70 years, and five times after a catheterization [7]; this suggests catheter-directed thrombectomy/thromboaspiration strategy coupled with fibrinolysis to be an alternative for patients with massive PE and high risk of hemorrhage. In this case, there was only one instance of bleeding from the puncture sites which did not require blood transfusion. An 8F catheter was used for thrombectomy and aspiration, even though there are other devices designed to administer fibrinolytics and to perform thromboaspiration, rotational thrombectomy, rheolytic disruption, ultrasound thrombolysis, or mixed techniques [8-12]. Although controlled trials would be necessary to evaluate their effectiveness and safety, it is unlikely that they be carried out due to the patients' serious condition and the differences among the techniques used in each hospital [8-12]. In addition, apixaban was selected because there is evidence for efficacy (non-inferiority) and security (significantly fewer major bleeding events) as compared with warfarin [13]. The apixabn dose was 10 mg BID for seven days (beginning the day after pulmonary thromboembolectomy), and 5 mg BID for three months. Taking into account that the optimal duration of anticoagulant therapy after the first episode of unprovoked venous thromboembolism is unclear, we decided 2.5 mg BID for one year; the course was uneventful [14].

## Conclusions

This elderly patient originally experienced an episode of intermediate-to-low-risk PE. However, it was misdiagnosed as ACS and it later evolved into a massive PE with acute *cor pulmonale* and high risk of bleeding. An echocardiogram on admission would have alerted to the correct diagnosis. The combined catheter-based pharmaco-mechanical strategy managed to reduce the clot burden, and the clinical condition improved significantly. Functional class, RV function and pulmonary artery pressure improved during follow-up. In massive PE, a combined fragmentation/thromboaspiration and catheter-directed fibrinolysis strategy may be useful to reduce the embolic load, improve RV function, and reduce mortality.
